# The influence of family socioeconomic status on college students’ mental health literacy: the chain mediating effect of parenting styles and interpersonal relationships

**DOI:** 10.3389/fpsyg.2024.1477221

**Published:** 2024-10-30

**Authors:** Jie Wu, Haoyuan Shen, Yunbo Shen, Xianglin Liao, Xianglian Yu

**Affiliations:** ^1^Psychology Department, Education College, Jianghan University, Wuhan City, China; ^2^Applied Psychology Department, Psychology College, Central China Normal University, Wuhan City, China

**Keywords:** family socioeconomic status, mental health literacy, parenting styles, interpersonal relationships, chain mediating role

## Abstract

**Introduction:**

With the increasing prevalence of mental health challenges among college students, understanding how family background and interpersonal dynamics affect mental health literacy is critical. This study examined the relationships between family socioeconomic status, mental health literacy, parenting styles, and interpersonal relationships among 1,107 college students.

**Methods:**

Using an online questionnaire, the study explored how family socioeconomic status, parenting styles, and interpersonal relationships influence mental health literacy.

**Results and discussion:**

The results showed that family socioeconomic status significantly and positively predicts mental health literacy. Both positive and negative parenting styles were found to partially mediate the relationship between family socioeconomic status and mental health literacy. Additionally, interpersonal relationships partially mediated this relationship. Finally, the study revealed that positive and negative parenting styles acted as sequential mediators between family socioeconomic status and mental health literacy through interpersonal relationships. These findings provide insights into the mechanisms by which family socioeconomic factors shape mental health literacy among college students.

## 1 Introduction

As the global socioeconomic landscape rapidly transforms, the pace of life has accelerated, bringing mental health issues to the forefront of public health concerns. The increasing prevalence of mental health disorders has garnered widespread attention. According to the World Health Organization (WHO), approximately 970 million people globally suffer from mental health disorders, with 42.3% of this population comprising young individuals (Freeman, 2022). Despite these alarming statistics, a significant number of individuals, particularly adolescents, do not seek treatment for mental health conditions (Özbıçakçı and Salkim, 2024). One of the key reasons for this is the lack of awareness regarding the symptoms of mental disorders and a generally low propensity to seek help (Tambling et al., 2023). These factors are closely linked to the concept of mental health literacy (MHL).

Mental health literacy has been found to correlate with various mental health disorders, such as depression (Blixen et al., 2016), anxiety (Li et al., 2013), and schizophrenia (Chen et al., 2017). In recent years, mental health literacy has gained increasing recognition in national policies. Since 2016, key documents like the Guiding Opinions on Strengthening Mental Health Services issued by the National Health Commission and other departments have highlighted the need to enhance national mental health literacy levels (Ming and Chen, 2020). Furthermore, the State Council’s Opinions on the Implementation of the Healthy China Initiative (2019) set a target to raise the population’s mental health literacy to 30% by 2030 (Jiang et al., 2021). Similarly, the WHO’s Mental Health Action Plan (2013–2030) underscores the importance of improving mental health and preventing mental disorders (Özparlak et al., 2023).

The concept of mental health literacy was first introduced by Jorm et al. (1997) as “knowledge and beliefs about mental disorders that aid in their recognition, management, and prevention.” In 2012, Jorm expanded this definition to encompass five components: knowledge of prevention, recognition, help-seeking, effective treatment strategies, self-help techniques, and psychological first aid skills (Jorm, 2012). A growing body of research suggests that socioeconomic status (SES) plays a significant role in shaping mental health literacy, with consistent findings across various studies (Jiang et al., 2021; Holman, 2015; Mahmoodi et al., 2022). Socioeconomic status is often conceptualized as a hierarchical measure of a family’s access to economic and social resources, reflecting an individual’s overall social position (Liu et al., 2024).

Parenting styles are a critical component of family dynamics and are defined by Darling and Steinberg (1993) as the attitudes parents display during everyday interactions with their children, creating distinct emotional climates. Studies indicate that parents from higher socioeconomic backgrounds tend to adopt more positive parenting styles, whereas those from lower socioeconomic backgrounds may resort to more negative approaches (Liu and Wu, 2022). Positive parenting styles have been shown to significantly enhance children’s mental health literacy (Chen, 2021).

The concept of interpersonal relationships, introduced by Mayo in 1933, refers to the mutual attraction or repulsion individuals experience during social interactions, influenced by their social experiences (Zhao and Dou, 2005). Research indicates that parental socioeconomic status affects children’s interpersonal relationships (Li and Guo, 2011), and positive interpersonal relationships are associated with higher levels of mental health literacy (Cui et al., 2020).

While extensive research has examined the influence of socioeconomic status on parenting styles, interpersonal relationships, and mental health, relatively few studies have comprehensively addressed mental health literacy. Most research has focused on individual variables without investigating the interplay between them. Numerous studies emphasize the importance of mental health literacy in promoting overall mental well-being (Bjornsen et al., 2017; Ming and Chen, 2020; Moss et al., 2022; Riiser et al., 2020; Schneider et al., 2021). The aim of the present study is to identify mediating variables such as parenting styles and interpersonal relationships and to explore how family socioeconomic status influences mental health literacy. This investigation aims to offer valuable insights and serve as a reference for future research in this area.

Specifically, this study seeks to examine the predictive effects of family socioeconomic status on parenting styles, interpersonal relationships, and mental health literacy. Additionally, it aims to explore how socioeconomic status, through the mediating roles of parenting styles and interpersonal relationships, affects mental health literacy. Based on the findings, relevant suggestions will be provided to enhance students’ mental health literacy and encourage greater attention to mental health literacy issues in both thought and action, contributing to healthy personal development.

### 1.1 Relationship between family socioeconomic status and mental health literacy

At the theoretical level, both the family stress model and family investment theory provide frameworks to understand the relationship between family socioeconomic status (SES) and mental health literacy. These theories posit that family economic conditions significantly influence children’s growth and development. Families with higher socioeconomic status can offer more developmental resources to their children, which has a positive impact on their mental health literacy (Jia and Zhu, 2021). Empirical research further supports this relationship, highlighting that educational attainment is a key factor in influencing individuals’ mental health literacy (Hou et al., 2019; Bindhim et al., 2024). Additionally, studies indicate that individuals with a higher perceived socioeconomic status tend to demonstrate better mental health literacy (Zhang et al., 2023). An international study also confirmed the positive association between family socioeconomic status and mental health literacy (Mahmoodi et al., 2022).

Given these findings, this study proposes Hypothesis 1: Family socioeconomic status is positively related to mental health literacy.

### 1.2 The mediating role of parenting styles

Parents with lower socioeconomic status are often observed to adopt more negative parenting styles. These approaches are characterized by minimal communication, stricter discipline, and a tendency to reject their children’s behaviors, demanding obedience and respect for authority (Zhang et al., 2022). Conversely, parents from higher socioeconomic backgrounds are more likely to employ positive parenting strategies, including open communication and emotional warmth (Liu and Wu, 2022; Zhao and Zeng, 2022). There is a significant negative correlation between socioeconomic status and negative parenting styles (Lin et al., 2023).

As primary caregivers, parents’ attitudes and behaviors have a profound influence on their children’s mental health literacy. Positive parenting styles, marked by warmth and responsiveness, have been shown to significantly improve children’s mental health literacy. In contrast, negative parenting styles, such as rejection or overprotection, are associated with lower levels of mental health literacy (Shan et al., 2014).

Based on these observations, this study proposes Hypothesis 2: Family socioeconomic status is positively associated with parenting style, and parenting style is positively associated with mental health literacy.

### 1.3 The mediating role of interpersonal relationships

Research indicates that socioeconomic status significantly influences children’s social relationships and their ability to form meaningful interpersonal connections (Ren, 2021; Jiang et al., 2018). College students from more affluent families typically demonstrate stronger interpersonal relationships. This may be due to their extroverted personalities, which facilitate engaging in social interactions and forming deep friendships. Conversely, students from economically disadvantaged families may tend to be more introverted, leading to difficulties in establishing close relationships (Xie, 2018).

Additionally, individuals with better interpersonal relationships are often found to have higher levels of mental health literacy. A study on mental health literacy among college students revealed a positive correlation between the strength of students’ interpersonal relationships and their mental health literacy (Cui et al., 2020; Zhang and Yin, 2023).

Thus, this study proposes Hypothesis 3: Family socioeconomic status is positively related to interpersonal relationships, and interpersonal relationships are positively related to mental health literacy.

### 1.4 The chain mediating role of parenting styles and interpersonal relationships

Existing research has consistently shown a significant relationship between parenting styles and the quality of parent–child interpersonal relationships (Yu et al., 2024). Huang (2010) demonstrated that children raised in households with positive parenting styles are more adept at forming strong interpersonal relationships. Conversely, negative parenting styles can lead to children rejecting social interactions and, in some cases, exhibiting aggressive or violent behaviors towards others. Similarly, Luo (2013) found that parenting styles significantly influence college students’ interpersonal relationships.

Furthermore, Tan (2021) showed that positive parenting styles foster an environment conducive to developing strong interpersonal relationships, whereas negative parenting styles tend to impede social engagement. This effect may be attributed to the emotional atmosphere established by parents: a warm and trusting environment promotes children’s willingness to engage with others, while a more restrictive or emotionally cold environment may foster distrust and feelings of inferiority, leading to social withdrawal (Li, 2021).

Based on these findings, this study proposes Hypothesis 4: Family socioeconomic status is positively associated with parenting style; parenting style is positively associated with interpersonal relationships; and interpersonal relationships are positively associated with mental health literacy.

### 1.5 The present study

A comprehensive review of both domestic and international literature reveals that most previous studies have primarily focused on the broad concept of mental health, with relatively limited attention given to the specific concept of mental health literacy. Furthermore, prior research has rarely explored the potential mediating variables that may influence the relationship between family socioeconomic status and mental health literacy. While many studies have examined the impact of socioeconomic status on parenting styles, interpersonal relationships, and mental health outcomes separately, few have examined these variables together in the context of mental health literacy.

Thus, the present study seeks to explore how family socioeconomic status affects mental health literacy through the mediating roles of parenting styles and interpersonal relationships. Specifically, this study aims to uncover the positive predictive effects of family socioeconomic status on parenting styles, interpersonal relationships, and mental health literacy. Furthermore, it will examine how family socioeconomic status influences mental health literacy indirectly through these mediating factors.

**Figure 1:**
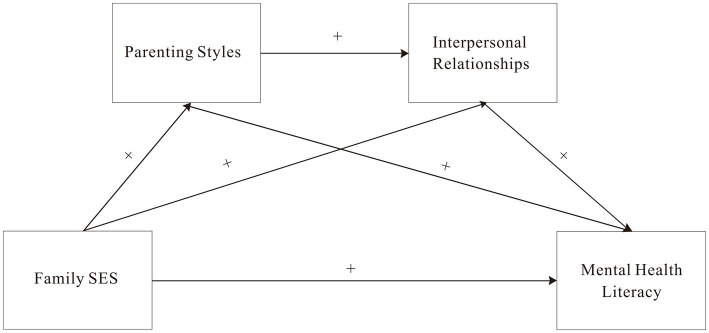
The chain mediation model. Family SES, family socioeconomic status. Any two of the four variables in the graph are positively correlated.

Building on the findings of previous research, the following hypotheses are proposed for this study (Figure 1):

*Hypothesis 1:* Family socioeconomic status is positively associated with mental health literacy.

*Hypothesis 2:* Family socioeconomic status is positively associated with parenting style, and parenting style is positively associated with mental health literacy.

*Hypothesis 3:* Family socioeconomic status is positively associated with interpersonal relationships, and interpersonal relationships are positively associated with mental health literacy.

*Hypothesis 4:* Family socioeconomic status is positively associated with parenting style, parenting style is positively associated with interpersonal relationships, and interpersonal relationships are positively associated with mental health literacy.

## 2 Materials and methods

### 2.1 Participants

This study employed a random sampling method to collect data from college students enrolled in various institutions across 11 provinces: Hubei, Guizhou, Sichuan, Henan, Fujian, Guangdong, Zhejiang, Jiangsu, Anhui, Beijing, and Liaoning. Prior approval was obtained from the respective universities, and written informed consent was secured from the participants. The consent form guaranteed anonymity and confidentiality, and participants were not required to sign or include their names. Students who agreed to participate completed the online questionnaire after reading the provided instructions. Upon completion, participants were offered snacks as a token of appreciation.

The study targeted college students, and a total of 1,200 questionnaires were collected. After a thorough screening process, 93 questionnaires were deemed invalid and excluded, resulting in 1,107 valid responses, representing a response rate of 92.25%. The sample included 359 male respondents (32.43%) and 748 female respondents (67.57%). Geographically, 641 participants (57.90%) were from rural areas, and 466 (42.10%) were from urban areas. In terms of academic year distribution, 551 respondents were freshmen (49.77%), 148 sophomores (13.37%), 304 juniors (27.46%), and 104 seniors (9.39%). As for academic disciplines, 455 participants were in liberal arts (41.10%), 538 in science and engineering (48.60%), and 114 in arts and physical education (10.30%). Notably, first-year students were the most represented group, while seniors were underrepresented, likely due to their lower presence on campus. Additionally, the sophomores had more classes, whereas the juniors had fewer, providing more opportunities to collect data from the latter group.

### 2.2 Measures

#### 2.2.1 Family socioeconomic status

In this study, the family socioeconomic status (SES) of university students was assessed using five indicators: annual family income, paternal education level, maternal education level, paternal occupation, and maternal occupation. These indicators were based on established methodologies from previous research (Bai, 2019; Tan and Kraus, 2015). Specifically:

Annual family income was measured on a 10-point scale, with higher scores indicating higher income.

Parental education levels were evaluated using a 6-point scale, where higher scores reflected higher levels of educational attainment.

Parental occupations were rated on a 7-point scale, with higher scores corresponding to more prestigious or higher-income occupations.

The overall SES score was calculated as the average of these five indicators, with higher scores reflecting a higher socioeconomic status. This approach provided a comprehensive assessment of socioeconomic factors that could influence the students’ educational and social experiences. The Cronbach’s alpha coefficient for this scale in the present study was 0.823, indicating good internal consistency.

#### 2.2.2 Mental health literacy

Mental health literacy was measured using the Adolescent Mental Health Literacy Assessment Scale, developed by Li et al. (2021). This scale comprises 22 items across four dimensions: knowledge, recognition, attitudes, and behaviors. Responses were recorded using a 5-point Likert scale with reverse scoring applied to specific items (2, 8, 13, 17, 18, and 19) to control for response biases. A higher total score on this scale indicated a higher level of mental health literacy. The scale demonstrated high reliability in this study, with a Cronbach’s alpha coefficient of 0.910, indicating excellent internal consistency.

#### 2.2.3 Parenting styles

To assess parenting styles, this study used the revised version of the Simplified Parenting Styles Questionnaire (S-EMBU-C), originally developed by Arrindell et al. (1999) and later modified by Jiang et al. (2010). The questionnaire consists of 42 items, with separate versions for the father’s and mother’s parenting styles, each containing 21 identical items. These items were distributed across three dimensions: emotional warmth, rejection, and overprotection. Responses were recorded on a 4-point Likert scale, with reverse scoring applied to item 23 in both versions to ensure accurate responses.

Following previous studies (Chen, 2020; Zhao and Zeng, 2022), the scores for each dimension across paternal and maternal versions were combined. The scores for overprotection and rejection were aggregated to form a measure of “negative parenting styles,” while the score for emotional warmth was used to define “positive parenting styles.” The scale demonstrated strong reliability in this study, with an overall Cronbach’s alpha coefficient of 0.822. The “negative parenting styles” subscale showed a coefficient of 0.930, and the “positive parenting styles” subscale had a coefficient of 0.910, both indicating excellent internal consistency.

#### 2.2.4 Interpersonal relationships

Interpersonal relationships were measured using the Comprehensive Interpersonal Diagnosis Scale developed by Zheng Richang. This scale consists of 28 items across four dimensions: conversation, social interaction and friendship, interpersonal etiquette, and interactions with friends of the opposite sex. Originally, responses of “yes” were scored as 1 point and “no” as 0 points, indicating greater interpersonal distress. However, for this study, the scoring system was reversed, with “yes” responses receiving 1 point and “no” responses receiving 2 points. A higher total score now indicated better interpersonal relationships. The scale demonstrated high reliability in this study, with a Cronbach’s alpha coefficient of 0.937.

### 2.3 Data analysis

As all the variables in this study were measured through self-reporting, there was a potential risk of common method bias. To assess and address this, Harman’s single-factor analysis was conducted on all variable items. Assuming no significant common method bias was detected, further statistical analyses were conducted using SPSS 27.0. These analyses included descriptive statistics, correlation analysis, difference testing, and regression analysis. Additionally, the PROCESS macro was utilized to test for mediation effects, providing a robust framework for understanding the relationships between the variables examined in this study.

## 3 Results

### 3.1 Common method bias test

Given that this study utilized self-reported questionnaires to assess the variables, there was a potential risk of common method bias. To address this, Harman’s single-factor test was employed, following the approach suggested by Zhou and Long (2004). The results revealed 17 factors with eigenvalues greater than 1, with the first factor accounting for 24.873% of the variance, which is below the critical threshold of 40%. These results suggest that common method bias was not a significant issue in this study.

### 3.2 Tests of difference between variables on demographic variables

The results of the difference tests are presented in Table 1. No significant differences were observed in family socioeconomic status (*t* = 0.127, *p* > 0.05), positive parenting styles (*t* = 0.596, *p* > 0.05), and negative parenting styles (*t* = 0.679, *p* > 0.05) between male and female participants. However, significant gender differences were found in interpersonal relationships (*t* = −2.003, *p* < 0.05, *d* = 0.133) and mental health literacy (*t* = −3.481, *p* < 0.001, *d* = 0.237), with female students scoring significantly higher than their male counterparts in both areas.

**Table 1:** Results of the test of variability of the variables on demographic variables (*N* = 1107).

Variables	Categories	Family SES	Positive parenting styles	Negative parenting styles	Interpersonal relationships	Mental health literacy
Sex	Male	3.789 ± 1.373	2.584 ± 0.690	1.963 ± 0.568	45.181 ± 8.336	77.663 ± 17.453
	Female	3.778 ± 1.343	2.558 ± 0.626	1.938 ± 0.584	46.226 ± 7.659	81.376 ± 14.713
	*t*	0.127	0.596	0.679	−2.003^*^	−3.481^***^
	*d*				0.133	0.237
Household registration	Rural	3.471 ± 1.323	2.503 ± 0.654	1.995 ± 0.602	45.541 ± 7.818	78.501 ± 15.662
	Urban	4.209 ± 1.274	2.654 ± 0.629	1.878 ± 0.538	46.363 ± 7.987	82.470 ± 15.579
	*t*	−9.299^***^	−3.841^***^	3.410^***^	−1.710	−4.172^***^
	*d*	0.566	0.234	0.204		0.254
Grade	Freshman	3.740 ± 1.313	2.585 ± 0.629	1.938 ± 0.584	45.935 ± 7.545	80.212 ± 15.211
	Sophomore	3.828 ± 1.412	2.546 ± 0.671	1.947 ± 0.540	46.277 ± 7.910	79.635 ± 16.266
	Junior	3.859 ± 1.421	2.582 ± 0.678	1.936 ± 0.573	45.865 ± 8.392	80.648 ± 16.280
	Senior	3.713 ± 1.268	2.457 ± 0.611	2.015 ± 0.621	45.144 ± 8.264	79.327 ± 16.322
	*F*	0.656	1.231	0.560	0.434	0.251
Major	Humanities and Social Sciences	3.819 ± 1.391	2.579 ± 0.674	1.927 ± 0.556	45.712 ± 8.427	79.222 ± 16.472
	Science and Engineering	3.725 ± 1.332	2.547 ± 0.618	1.944 ± 0.591	46.022 ± 7.362	81.290 ± 15.129
	Arts and Physical Education	3.902 ± 1.289	2.608 ± 0.671	2.030 ± 0.608	45.947 ± 8.195	78.684 ± 15.357
	*F*	1.090	0.552	1.449	0.194	2.703

^*^p < 0.05, ^**^p < 0.01, ^***^p < 0.001.

Regarding residential registration, there were no significant differences in interpersonal relationships (*t* = −1.710, *p* > 0.05). However, significant differences were found for family socioeconomic status (*t* = −9.299, *p* < 0.001, *d* = 0.566), positive parenting styles (*t* = −3.841, *p* < 0.001, *d* = 0.234), negative parenting styles (*t* = 3.410, *p* < 0.001, *d* = 0.204), and mental health literacy (*t* = −4.172, *p* < 0.001, *d* = 0.254). Specifically, students from urban areas scored significantly higher in family socioeconomic status, positive parenting styles, and mental health literacy compared to their rural counterparts. In contrast, students from urban areas scored significantly lower in negative parenting styles than those from rural areas.

There were no significant differences observed across academic years in terms of family socioeconomic status (*F* = 0.656, *p* > 0.05), positive parenting styles (*F* = 1.231, *p* > 0.05), negative parenting styles (*F* = 0.560, *p* > 0.05), interpersonal relationships (*F* = 0.434, *p* > 0.05), and mental health literacy (*F* = 0.251, *p* > 0.05). Similarly, no significant differences were found across academic disciplines for family socioeconomic status (*F* = 1.090, *p* > 0.05), positive parenting styles (*F* = 0.552, *p* > 0.05), negative parenting styles (*F* = 1.449, *p* > 0.05), interpersonal relationships (*F* = 0.194, *p* > 0.05), and mental health literacy (*F* = 2.703, *p* > 0.05).

In conclusion, gender and household registration were identified as significant factors and thus were included as control variables in subsequent analyses.

### 3.3 Descriptive statistics and correlation analysis

The descriptive statistics and results of the correlation analysis for each variable are presented in Table 2. The findings indicate that positive parenting styles (*r* = 0.542, *p* < 0.01), interpersonal relationships (*r* = 0.462, *p* < 0.01), and mental health literacy (*r* = 0.456, *p* < 0.01) are significantly and positively correlated with family socioeconomic status. In contrast, negative parenting styles showed significant negative correlations with family socioeconomic status (*r* = −0.380, *p* < 0.01), positive parenting styles (*r* = −0.485, *p* < 0.01), interpersonal relationships (*r* = −0.438, *p* < 0.01), and mental health literacy (*r* = −0.518, *p* < 0.01).

**Table 2:** Descriptive statistics and correlation analysis (*N* = 1107).

Variables	1	2	3	4	5
1. Family SES	1				
2. Positive parenting styles	0.542^**^	1			
3. Negative parenting Styles	−0.380^**^	−0.485^**^	1		
4. Interpersonal relationships	0.462^**^	0.575^**^	−0.438^**^	1	
5. Mental health literacy	0.456^**^	0.481^**^	−0.518^**^	0.448^**^	1
*M*	3.782	2.567	1.946	45.887	80.172
*SD*	1.352	0.647	0.579	7.896	15.742

^*^p < 0.05, ^**^p < 0.01, ^***^p < 0.001.

Additionally, positive parenting styles (*r* = 0.481, *p* < 0.01) and interpersonal relationships (*r* = 0.448, *p* < 0.01) were significantly and positively associated with mental health literacy. A significant positive correlation was also observed between positive parenting styles and interpersonal relationships (*r* = 0.481, *p* < 0.01).

### 3.4 Chain mediation effect testing

#### 3.4.1 Examination of the chain mediation effects of positive parenting styles and interpersonal relationships

First, Model 6 of the PROCESS macro in SPSS was employed to examine the chain mediation effects. In this model, family socioeconomic status was set as the independent variable, mental health literacy as the dependent variable, and positive parenting styles and interpersonal relationships as the mediating variables. Gender and household registration were included as control variables. The results are summarized in Table 3.

**Table 3:** Chain mediation regression analysis of positive parenting styles and interpersonal relationships.

Regression equation (*n* = 1107)		Fit Indices			Standardized regression coefficients	
Outcome variable	Predictor variable	*R*	*R^2^*	*F*	*β*	*t*
Mental health literacy	Family SES	0.470	0.221	104.125	0.458	16.601^***^
	Sex				0.113	4.227^***^
	Household registration				−0.007	−0.234
Positive parenting styles	Family SES	0.544	0.295	154.139	0.551	20.993^***^
	Sex				−0.014	−0.562
	Household registration				−0.033	−1.243
Interpersonal relationships	Family SES	0.610	0.372	163.480	0.232	7.913^***^
	Positive parenting styles				0.459	16.140^***^
	Sex				0.076	3.175^**^
	Household registration				−0.069	−2.771^**^
Mental health literacy	Family SES	0.568	0.323	105.077	0.229	7.314^***^
	Positive parenting styles				0.246	7.477^***^
	Interpersonal relationships				0.194	6.187^***^
	Sex				0.103	4.112^***^
	Household registration				0.018	0.687

All variables included in the analysis were standardized. ^*^p < 0.05, ^**^p < 0.01, ^***^p < 0.001.

The analysis revealed that family socioeconomic status significantly and positively predicts mental health literacy (*β* = 0.458, *t* = 16.601, *p* < 0.001). Even after the inclusion of the two mediating variables, family socioeconomic status remained a significant positive predictor of mental health literacy (*β* = 0.229, *t* = 7.314, *p* < 0.001). Additionally, family socioeconomic status significantly and positively predicts both positive parenting styles (*β* = 0.551, *t* = 20.993, *p* < 0.001) and interpersonal relationships (*β* = 0.232, *t* = 7.913, *p* < 0.001). Furthermore, positive parenting styles significantly predict interpersonal relationships (*β* = 0.459, *t* = 16.140, *p* < 0.001) and mental health literacy (*β* = 0.246, *t* = 7.477, *p* < 0.001). Similarly, interpersonal relationships significantly predict mental health literacy (*β* = 0.194, *t* = 6.187, *p* < 0.001).

Next, the Bootstrap method was used to further analyze the mediation effects of positive parenting styles and interpersonal relationships between family socioeconomic status and mental health literacy, with the results presented in Table 4. The Bootstrap 95% confidence interval for the direct effect of family socioeconomic status on mental health literacy did not include zero, confirming the significance of the direct effect (effect size = 0.229), which accounts for 49.99% of the total effect. Additionally, the Bootstrap 95% confidence intervals for the mediation effects of positive parenting styles and interpersonal relationships between family socioeconomic status and mental health literacy also did not include zero, demonstrating the significance of these indirect effects. The total effect size of the indirect effects was 0.229, accounting for 50.01% of the total effect.

**Table 4:** Direct, indirect, and total effects of the hypothesized model.

Model pathways	Effects	Boot SE	Boot LLCI	Boot ULCI	Proportion of effects
Direct effect					
Family SES → Mental health literacy	0.229	0.031	0.168	0.291	49.99%
Indirect effects	0.229	0.022	0.187	0.274	50.01%
Family SES → Positive parenting styles → Mental health literacy	0.135	0.022	0.091	0.178	29.52%
Family SES → Interpersonal relationships → Mental health literacy	0.045	0.009	0.028	0.065	9.80%
Family SES → Positive parenting styles → Interpersonal relationships → Mental health literacy	0.049	0.010	0.031	0.069	10.69%
Total effect	0.458	0.028	0.404	0.513	

The findings indicate that positive parenting styles and interpersonal relationships serve as partial mediators between family socioeconomic status and mental health literacy, with three distinct mediation paths contributing to the overall effect:

1. Family socioeconomic status → Positive parenting styles → Mental health literacy, with an effect size of 0.135, accounting for 29.52% of the total effect.
2. Family socioeconomic status → Interpersonal relationships → Mental health literacy, with an effect size of 0.045, accounting for 9.80% of the total effect.
3. Family socioeconomic status → Positive parenting styles → Interpersonal relationships → Mental health literacy, with an effect size of 0.049, accounting for 10.69% of the total effect.

**Figure 2:**
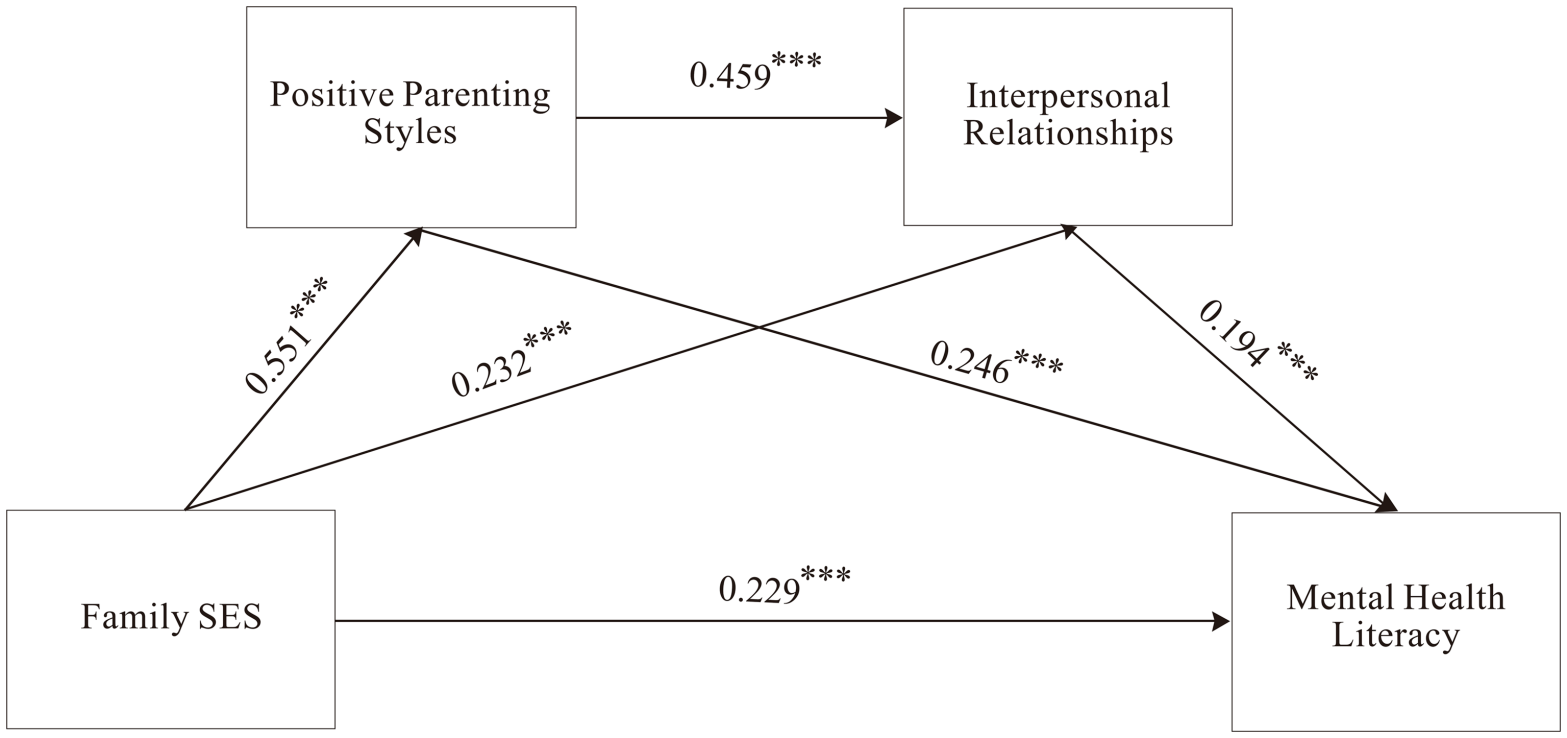
Diagram of the chain mediation model between positive parenting styles and interpersonal relationships. Positive parenting styles and interpersonal relationships as chain mediators between family socioeconomic status and mental health literacy. *p < 0.05, **p < 0.01, ***p < 0.001.

The final mediation model is illustrated in Figure 2.

#### 3.4.2 Examination of the chain mediation effects of negative parenting styles and interpersonal relationships

To examine the chain mediation effects, Model 6 of the PROCESS macro in SPSS was utilized. Family socioeconomic status was treated as the independent variable, mental health literacy as the dependent variable, and negative parenting styles and interpersonal relationships as mediating variables. Gender and residence were included as control variables, as presented in Table 5.

**Table 5:** Chain mediation regression analysis of negative parenting styles and interpersonal relationships.

Regression equation (*n* = 1107)		Fit Indices			Standardized regression coefficients	
Outcome variable	Predictor variable	*R*	*R^2^*	*F*	*β*	*t*
Mental health literacy	Family SES	0.470	0.221	104.125	0.458	16.601^***^
	Sex				0.113	4.227^***^
	Household registration				−0.007	−0.234
Negative parenting styles	Family SES	0.381	0.145	62.466	−0.382	−13.198^***^
	Sex				−0.022	−0.793
	Household registration				0.004	0.139
Interpersonal relationships	Family SES	0.551	0.304	120.148	0.369	13.115^***^
	Negative parenting styles				−0.305	−11.225^***^
	Sex				0.063	2.487^*^
	Household registration				−0.083	−3.160^**^
Mental health literacy	Family SES	0.617	0.380	135.192	0.239	8.391^***^
	Negative parenting styles				−0.346	−12.767^***^
	Interpersonal relationships				0.180	6.321^***^
	Sex				0.093	3.878^***^
	Household registration				0.010	0.403

All variables included in the analysis were standardized. ^*^p < 0.05, ^**^p < 0.01, ^***^p < 0.001.

The results indicated that family socioeconomic status significantly and positively predicts mental health literacy (*β* = 0.458, *t* = 16.601, *p* < 0.001). After including the two mediating variables, family socioeconomic status remained a significant positive predictor of mental health literacy (*β* = 0.239, *t* = 8.391, *p* < 0.001). Additionally, family socioeconomic status significantly and negatively predicts negative parenting styles (*β* = −0.382, *t* = −13.198, *p* < 0.001), while it significantly and positively predicts interpersonal relationships (*β* = 0.369, *t* = 13.115, *p* < 0.001). The analysis further revealed that negative parenting styles significantly and negatively predict both interpersonal relationships (*β* = −0.305, *t* = −11.225, *p* < 0.001) and mental health literacy (*β* = −0.346, *t* = −12.767, *p* < 0.001). In contrast, interpersonal relationships significantly and positively predict mental health literacy (*β* = 0.180, *t* = 6.321, *p* < 0.001).

Subsequently, the Bootstrap method was employed to analyze the mediation effects of negative parenting styles and interpersonal relationships between family socioeconomic status and mental health literacy, with the results displayed in Table 6. The Bootstrap 95% confidence interval for the direct effect of family socioeconomic status on mental health literacy did not include 0, confirming a significant direct effect (effect size = 0.239), accounting for 52.19% of the total effect. Moreover, the Bootstrap 95% confidence intervals for the mediation effects of negative parenting styles and interpersonal relationships between family socioeconomic status and mental health literacy also did not include 0, indicating that these mediation effects are significant. The total indirect effect size was 0.219, accounting for 47.81% of the total effect.

**Table 6:** Direct, indirect, and total effects of the hypothesized model.

Model pathways	Effects	Boot SE	Boot LLCI	Boot ULCI	Proportion of effects
Direct effect					
Family SES → Mental health literacy	0.239	0.029	0.183	0.295	52.19%
Indirect effects	0.219	0.020	0.182	0.258	47.81%
Family SES → Negative parenting styles → Mental health literacy	0.132	0.015	0.104	0.162	28.80%
Family SES → Interpersonal relationships → Mental health literacy	0.066	0.013	0.043	0.092	14.44%
Family SES → Negative parenting styles → Interpersonal relationships → Mental health literacy	0.021	0.004	0.014	0.029	4.56%
Total effect	0.458	0.028	0.404	0.513	

The findings indicate that negative parenting styles and interpersonal relationships serve as partial mediators between family socioeconomic status and mental health literacy, with three influential pathways:

1. Family socioeconomic status → Negative parenting styles → Mental health literacy, with an effect size of 0.132, accounting for 28.80% of the total effect.
2. Family socioeconomic status → Interpersonal relationships → Mental health literacy, with an effect size of 0.066, accounting for 14.44% of the total effect.
3. Family socioeconomic status → Negative parenting styles → Interpersonal relationships → Mental health literacy, with an effect size of 0.021, accounting for 4.56% of the total effect.

**Figure 3:**
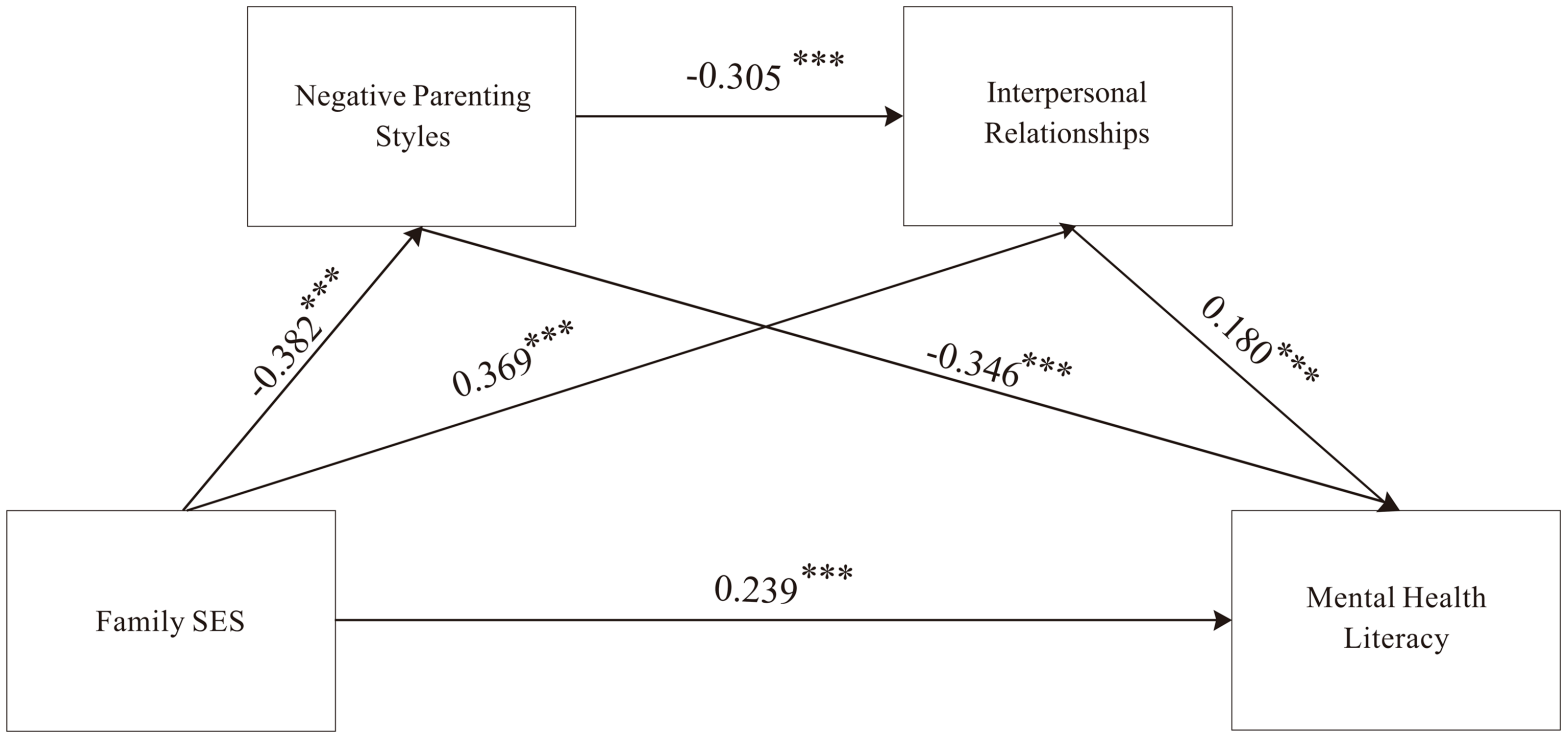
Diagram of the chain mediation model between negative parenting styles and interpersonal relationships. Negative parenting styles and interpersonal relationships as chain mediators between family socioeconomic status and mental health literacy. *p < 0.05, **p < 0.01, ***p < 0.001.

The final model depicting these mediation effects is illustrated in Figure 3.

## 4 Discussion

### 4.1 Comparative analysis of demographic variables across all factors

#### 4.1.1 Family socioeconomic status

The results indicate no significant differences in family socioeconomic status based on gender, grade level, or major among college students. However, students with urban household registrations significantly outperform their rural counterparts in terms of socioeconomic status, which is consistent with previous research (Yang, 2024). This disparity likely stems from the higher levels of educational attainment and greater annual family incomes typically seen in urban households. One major factor limiting rural workers’ access to the primary job market is the household registration system, where urban workers enjoy near-exclusive access to both the primary and secondary markets (Wang, 2023). As a result, university students with urban household registration, along with their families, benefit from more favorable occupational opportunities and economic conditions.

#### 4.1.2 Mental health literacy

Significant differences in mental health literacy were observed based on gender and household registration. Female students and those from urban areas exhibited higher levels of mental health literacy, likely due to better access to mental health resources and information via the internet (Khatib and Abo-Rass, 2022). This finding aligns with prior studies (Soria-Martínez et al., 2024; Wang J. et al., 2024). Additionally, men may be more prone to using general terms like “mental illness” rather than specific terms such as “depression” when identifying mental health issues, leading to less accurate recognition of specific disorders (Singh et al., 2022). For those living in rural or remote areas, access to mental health services remains limited, contributing to generally lower levels of mental health literacy (Li et al., 2023). Notably, no significant differences in mental health literacy were found across different grade levels or academic majors.

#### 4.1.3 Parenting styles

The analysis shows no significant differences in positive or negative parenting styles based on gender, grade level, or major. However, there were notable differences based on household registration, with urban parents, likely due to their higher education levels, more often employing positive parenting practices (He, 2020). In contrast, parents in rural families are more inclined to use overprotective parenting methods, stemming from fears that their children may suffer harm, particularly to their self-esteem and confidence (Li, 2021).

#### 4.1.4 Interpersonal relationships

While no significant differences in interpersonal relationships were found based on household registration, grade level, or major, gender differences were evident. Female students typically engage in more diverse social interactions, leading to broader social networks (Wang, 2022). This difference may be explained by gender-based social tendencies, with girls often being more collectivist and boys more individualistic, which may contribute to stronger interpersonal relationships among female students (Bi et al., 2016).

### 4.2 Relationship between family socioeconomic status and mental health literacy

The results of this study indicate that family socioeconomic status significantly and positively predicts mental health literacy, consistent with findings from both domestic and international scholars (Abo-Rass et al., 2024; Jiang et al., 2021; Holman, 2015). This validation supports the first hypothesis of this study.

College students’ socioeconomic status is often linked to their parents’ level of education. Higher parental education correlates with greater access to mental health knowledge and more comprehensive mental health education. In daily family interactions, parents with higher educational attainment have more opportunities to disseminate mental health information to their children, offering additional support and resources that can enhance their children’s mental health literacy (Zhang and Yin, 2023). Furthermore, family income is also associated with mental health literacy (Mahmoodi et al., 2022). Regarding help-seeking behaviors, which are closely related to mental health literacy (Jia et al., 2023; Ren et al., 2020; Xiao and Zheng, 2024), adolescents from higher socioeconomic backgrounds are more inclined to seek help (Abo-Rass et al., 2024). Other studies have shown that adolescents from lower-income families tend to have lower levels of mental health literacy (Lima et al., 2024). Additionally, adolescents from wealthier families are more likely to pursue mental health services, whereas lower-income peers may encounter parental reluctance in prioritizing such services (Goosby, 2007). These dynamics significantly influence the willingness of children to seek mental health assistance, ultimately affecting their mental health literacy.

### 4.3 The mediating role of parenting styles

The findings of this study suggest that both positive and negative parenting styles partially mediate the relationship between family socioeconomic status and mental health literacy, consistent with previous research (Chen, 2021; Zhao and Zeng, 2022). This validates the second hypothesis of this study.

In Chinese society, family socioeconomic status is largely shaped by parents’ educational attainment. Traditional cultural influences often result in subtle emotional expressions from parents (Chen, 2024). However, as parental education levels increase, parents tend to better understand the importance of adopting positive parenting styles, which foster emotional warmth and contribute to their children’s healthy development (Chen, 2024). Research shows that parents from higher socioeconomic backgrounds are more likely to employ positive parenting strategies with their children (Yang and Zhao, 2020). A meta-analysis conducted abroad also confirms a positive correlation between family socioeconomic status and positive parenting styles, as well as a negative correlation between socioeconomic status and negative parenting styles (Ayoub and Bachir, 2023).

From the perspective of mental health literacy, children raised under negative parenting styles are more prone to experiencing mental health issues (Feng et al., 2023). This can hinder their ability to develop positive coping mechanisms, subsequently lowering their mental health literacy.

### 4.4 The mediating role of interpersonal relationships

The results of this study also suggest that interpersonal relationships partially mediate the relationship between family socioeconomic status and mental health literacy, consistent with previous research (Xie, 2018; Zhang and Yin, 2023). This confirms the third hypothesis of this study.

Adolescents from lower socioeconomic backgrounds often display higher levels of pessimism and hostility due to the challenging environments in which they grow up (Luo and Li, 2015). As a result, they are less likely to initiate communication with others, making it difficult to form positive interpersonal relationships. Scholars argue that adolescents from higher socioeconomic backgrounds have more time and energy to engage in social interactions, which helps them establish and maintain positive relationships (Li et al., 2022).

From the perspective of mental health literacy, interpersonal relationships can significantly shape individuals’ perceptions and attitudes towards mental illness (Huggard and O’Connor, 2023). Students with strong interpersonal relationships tend to have more robust social networks, allowing them to engage in more frequent interpersonal communication. This, in turn, provides greater access to diverse mental health knowledge across different social spheres, ultimately enhancing their mental health literacy (Zhang and Yin, 2023).

### 4.5 The chain mediating role of parenting styles and interpersonal relationships

The results of this study indicate that both positive and negative parenting styles, along with interpersonal relationships, play a chain-mediated role between family socioeconomic status and mental health literacy. This finding is consistent with prior research (Tan, 2021), thus validating the fourth hypothesis of this study.

Parents with high socioeconomic status tend to adopt positive parenting styles, whereas parents with lower socioeconomic status are more likely to employ negative parenting styles (Ayoub and Bachir, 2023). One possible explanation is that parents with higher socioeconomic status are more adept at using positive parenting strategies to meet their children’s growing need for autonomy, particularly during their secondary school years (Butler and Le, 2018).

When parents treat their children with warmth, they engage in more frequent and positive emotional exchanges. This interaction not only strengthens the harmony of the parent–child relationship but also fosters the development of harmonious interpersonal relationships between the children and others. Children raised in such warm environments often develop greater empathy and a stronger ability to consider others’ perspectives, which helps them form positive interpersonal relationships (Duan et al., 2023). Conversely, parents who employ negative parenting styles such as rejection, denial, or punishment may find their children struggling with interpersonal relationship issues (Guo Y. et al., 2024). Chronic rejection by parents can lead to feelings of inferiority and self-doubt, making children’s self-esteem more fragile, which may result in social withdrawal and excessive wariness in interpersonal interactions, ultimately contributing to interpersonal difficulties (Wang, 2022). On the other hand, studies have shown that positive parenting styles foster good relationships between siblings, while negative parenting styles hinder such relationships (Krejcova et al., 2023). Additionally, relationships play a key role in positive mental health literacy (Carvalho et al., 2022); thus, strong interpersonal relationships contribute to higher levels of mental health literacy. Current research suggests that poor interpersonal relationships may hinder individuals from seeking help, which in turn negatively impacts their mental health literacy (Guo S. et al., 2024).

### 4.6 Relationship between the four variables

Existing research suggests that family socioeconomic status can influence mental health literacy in relation to income (Mahmoodi et al., 2022) and educational attainment (Zhang and Yin, 2023), supporting the notion that family socioeconomic status is positively related to mental health literacy. Additionally, highly educated parents tend to adopt more positive parenting styles, granting their children more autonomy and using less control compared to parents with lower educational attainment (Butler and Le, 2018). Lower socioeconomic status, on the other hand, has been linked to poorer interpersonal relationships (Park et al., 2023). Given that parental education is a core component of family socioeconomic status, it is expected that family socioeconomic status is positively correlated with both parenting styles and interpersonal relationships.

Furthermore, positive parenting styles are associated with better mother–child relationships (Shieh and Tsai, 2022), improved sibling relationships (Krejcova et al., 2023), and enhanced mental health literacy (Shan et al., 2014). Research also indicates that parent–child relationships significantly predict children’s mental health literacy (Wang X. et al., 2024), suggesting that interpersonal relationships are positively correlated with mental health literacy.

## 5 Implications

This study provides valuable insights into the influence of family socioeconomic status on mental health literacy, highlighting the complex mechanisms through which socioeconomic factors impact mental well-being. As a result, educational institutions, particularly universities, should broaden their educational mandates to include initiatives aimed at enhancing mental health literacy, especially for students from lower socioeconomic backgrounds. Such initiatives would ensure that all students, regardless of their family’s economic standing, attain a sufficient level of mental health literacy.

From a parental perspective, given the findings that parenting styles significantly influence both interpersonal relationships and mental health literacy, it is crucial for parents to actively foster their children’s development in these areas. In everyday interactions, parents should adopt positive parenting practices, addressing their children’s academic and emotional needs attentively. They should also share mental health knowledge and provide guidance on seeking psychological help when necessary. Furthermore, parents should cultivate a warm and supportive home environment, focusing on strengthening their children’s interpersonal relationships to support their overall development.

For college students, improving mental health literacy should be a personal priority. This involves cultivating strong interpersonal relationships, expanding social networks, and actively participating in mental health education initiatives. By learning about mental health and understanding mental disorders, students can enhance their literacy and overall well-being. Engaging with diverse sources of information is crucial for developing a well-rounded understanding of mental health.

## 6 Limitations and suggestions for further studies

This study was conducted with a relatively small and homogeneous sample of undergraduate students, with limited age variation. Moreover, the reliance on self-report questionnaires may have introduced social desirability bias, potentially skewing participants’ responses away from their true perspectives. Additionally, the study focused solely on the effects of family socioeconomic status on mental health literacy without considering subjective socioeconomic status as a variable and included a limited set of control variables.

To address these limitations, future research could broaden the sample to include graduate or high school students, enhancing the generalizability and diversity of the findings. Methodologically, incorporating cognitive neuroscience techniques, such as electroencephalography (EEG), could provide more objective data and increase the scientific rigor of the results. Furthermore, adding subjective socioeconomic status as a variable would allow for a more comprehensive analysis of its impact on mental health literacy. Future research should also explore additional mediating and moderating variables that influence the relationship between family socioeconomic status and mental health literacy and consider including more control variables in the analysis.

## 7 Conclusion

This study explored the relationship between family socioeconomic status and mental health literacy among college students, focusing on the mediating roles of parenting styles and interpersonal relationships. The key findings are as follows:

1. Family socioeconomic status significantly predicts mental health literacy.
2. Both positive and negative parenting styles act as partial mediators between family socioeconomic status and mental health literacy.
3. Interpersonal relationships also serve as partial mediators, affecting the influence of family socioeconomic status on mental health literacy.
4. A chain mediation effect exists, where both types of parenting styles influence mental health literacy through their impact on interpersonal relationships.

## Data Availability

The raw data supporting the conclusions of this article will be made available by the authors, without undue reservation.
